# Primary scrotal melanoma: it is time to destigmatize genital lesions^☆^^☆☆^

**DOI:** 10.1016/j.abd.2018.12.005

**Published:** 2019-11-02

**Authors:** Ezgi Özkur, İlknur Kıvanç Altunay

**Affiliations:** Department of Dermatology, Şişli Etfal Training and Research Hospital, University of Health Sciences, Istanbul, Turkey

**Keywords:** Melanoma, Scrotum, Urogenital neoplasms

## Abstract

Primary male genital melanomas are very rare; they are associated with high mortality and late detection. Scrotal melanoma is the least common presentation and only 23 cases have been reported. Herein, the authors present a 30-year-old patient with stage IIIC (T4b, N2a, M0) scrotal melanoma in order to report the characteristics, treatment, and outcome, as well as to emphasize the importance of examination of the genitals, education of patients about self-examination and destigmatizing genital lesions to increase the likelihood of earlier detection.

## Introduction

Primary male genital melanomas are very rare; among them, scrotal melanoma is the least common, and only 23 cases have been reported so far in medical literature.^1^ Genitourinary (GU) melanomas can arise from cutaneous (*i.e*., scrotum, penile shaft) or mucosal (urothelial) surfaces. Primary GU melanomas has been estimated to represent less than 1% of all melanomas.^2^ Unfortunately, these patients usually present with late-stage disease and poorer outcomes.^3^ Herein, the authors report a patient with scrotal melanoma to demonstrate patient characteristics, treatment, and outcome, as well as to reinforce the importance of examination of the genitals.

## Case report

**Figure 1:**
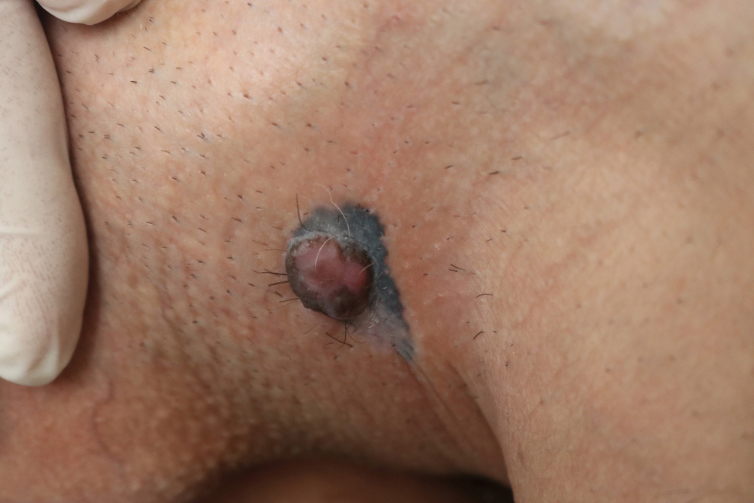
Blue-black colored melanocytic macule with a pinkish tumor on top of it.

**Figure 2:**
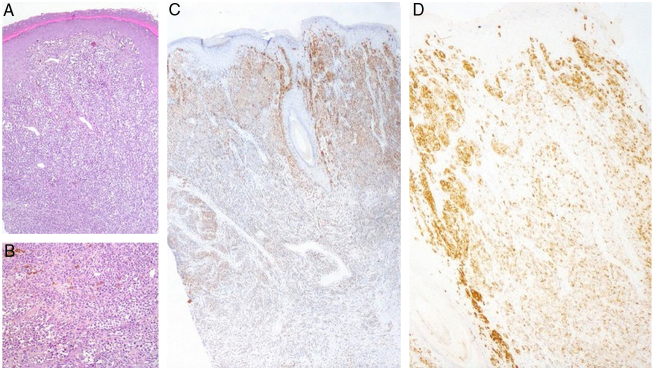
(A) Hematoxylin & eosin staining (x20) found a well-circumscribed, non-inflammatory dermal mass with atypical melanocytes, and no epidermal connection. (B) Hematoxylin & eosin staining (x40) showed atypical melanocytes. (C) Melan-A immunohistochemical staining. (D) HMB-45 immunohistochemical staining.

A 30-year-old man was attended to by the clinic with a history of black-colored lesion on his scrotum, which had started a year previously and gradually enlarged. The patient reported that he had waited a year to heal spontaneously because of being embarrassed to have a genital examination. He had no systemic symptoms or medical history of any illness. On examination, 5-cm-diameter blue-black colored macule was discovered on the left side of scrotum, with a 3-cm-diameter, non-tender, well-circumscribed pink nodule on top of it (Fig. 1). His physical examination and routine laboratory investigations were otherwise unremarkable. The authors performed a 4-mm skin biopsy taken from the nodule. Histopathology confirmed the diagnosis of malign melanoma and showed a dermal mass with atypical nested melanocytes, with frequent mitotic figures that were strongly positive for S-100 and melan-A (Fig. 2). Wide local excision with 2-cm surgical margins was performed. The histopathological analysis found pathological stage T4b, 6-mm Breslow depth, Clark level IV, 6 mitosis/mm^2^ nodular melanoma with ulceration, without vascular invasion, lymphatic invasion, or microsatellitosis, and the tumor was also positive for the BRAF V600 mutation. In sentinel lymph node biopsy, two-node metastasis was detected without extracapsular extension. Two weeks later, elective dissection of the regional lymph nodes was performed, but the nodes were tumor-free. PET CT scan found no further evidence of metastatic disease. His disease was defined as stage IIIC (T4b, N2a, M0) based on the American Joint Commission Cancer staging guideline for melanoma. He was referred to the medical oncology department and started high-dose intravenous interferon α-2b (20,000,000 international units [IU]/m^2^, five days per week) for one month, followed by subcutaneous injections (10,000,000 IU/m^2^, three times per week). The patient underwent clinical examination every three months, and neither recurrence nor distance metastasis were detected in first year of his treatment.

## Discussion

Scrotal melanomas are associated with high mortality and late presentation. Zikry et al.^1^ conducted a systematic literature review, yielding 23 cases with scrotal melanoma and found that tumor presented as stage I/II disease 18.75% of the time, stage III 56.3% of the time, and stage IV 25% of the time, whereas typical cutaneous melanoma presents as stage I/II disease 84% of the time. They also reported that half of patients experienced recurrence of their disease. Sánchez-Ortiz et al.^2^ reported that even though patients who undergo scrotectomy achieve effective local control, they may remain at significant risk for regional recurrence. The same researchers suggested that wide local excision and prophylactic, modified lymphadenectomy should be performed in patients with scrotal melanoma with a Breslow depth of 1 mm or greater, ulceration, or Clark level IV or V. The present authors could not start BRAF inhibitors in this patient because insurance policies only pay treatment costs for targeted therapies in distant metastatic disease.

As dermatologists, current evidence shows that primary GU melanomas detected in later stages have poorer outcomes, so it is very important for dermatologists to perform detailed genital exams. The incidence, clinicopathologic characteristics, treatment, and survival outcomes are not yet very well-described for primary GU melanoma.^4^ Thus, it is uncertain whether the advanced stage of GU melanomas at presentation is because of late detection or the intrinsic characteristics of the tumor.

Houston et al. suggested that patients are more likely to agree to a total body skin examination when clinicians respect the patient's preference for the physician's gender.^5^ Zikry et al.^6^ recommended, concerning the positioning of patients during these genital exams, that male genitalia can be examined with the patient standing and the physician seated on a stool in front of the patient. Also, they added that male genital skin exams should include retraction of the foreskin of the penis and inspection of the dorsal and ventral penis, as well as all aspects of the scrotum. Additionally, educating patients about self-examination and destigmatizing genital lesions will increase the likelihood of earlier detection and better survival rates.

## Financial support

None declared.

## Author's contribution

Ezgi Ozkur: Conception and planning of the study; elaboration and writing of the manuscript; obtaining, analyzing and interpreting the data; intellectual participation in propaedeutic and/or therapeutic conduct of the cases studied; critical review of the literature.

İlknur Kıvanç Altunay: Approval of the final version of the manuscript; critical review of the manuscript.

## Conflicts of interest

None declared.
